# Supplementary material for: Investigating whether smoking and alcohol behaviours influence risk of type 2 diabetes using a Mendelian randomisation study
Source: Sci Rep. 2025 Mar 7;15:7985. doi: 10.1038/s41598-025-90437-x (PMC11889105; doi:10.1038/s41598-025-90437-x)
Supplement: Supplementary file 2 — Supplementary Material 2 [file 41598_2025_90437_MOESM2_ESM.docx]

**STROBE-MR checklist of recommended items to address in reports of Mendelian randomization studies**^1^ ^2^

| **Item No.** | **Section** | **Checklist item** | **Page No.** | **Relevant text from manuscript** |
| --- | --- | --- | --- | --- |
| 1 | **TITLE and ABSTRACT** | Indicate Mendelian randomization (MR) as the study’s design in the title and/or the abstract if that is a main purpose of the study | 1 to 2 | Title: Investigating whether smoking and alcohol behaviours influence risk of type 2 diabetes using a Mendelian randomisation study  This is also mentioned throughout the abstract. |
|  | **INTRODUCTION** |  |  |  |
| 2 | **Background** | Explain the scientific background and rationale for the reported study. What is the exposure? Is a potential causal relationship between exposure and outcome plausible? Justify why MR is a helpful method to address the study question | 3 to 7 | Exposures are alcohol and smoking. We discuss the background evidence suggesting an association here and discuss relevant MR studies and their limitations. We introduce MR as a useful tool to examine this further including a range of outcomes related to T2D and using the most recent GWAS for exposures and outcomes. |
| 3 | **Objectives** | State specific objectives clearly, including pre-specified causal hypotheses (if any). State that MR is a method that, under specific assumptions, intends to estimate causal effects | 5 to 7 | “The aim of this study was to use MR to explore the effects of lifetime smoking and alcohol consumption (drinks per week) on T2D risk and underlying glycaemic traits”.  We discuss MR and its purpose as well as assumptions (Box 1). |
|  | **METHODS** |  |  |  |
| 4 | **Study design and data sources** | Present key elements of the study design early in the article. Consider including a table listing sources of data for all phases of the study. For each data source contributing to the analysis, describe the following: |  |  |
|  | a) | Setting: Describe the study design and the underlying population, if possible. Describe the setting, locations, and relevant dates, including periods of recruitment, exposure, follow-up, and data collection, when available. | 18 to 20 | GWAS included are discussed including sample size, cohort, ancestry for two-sample MR. Details on accessing these and further details on measures are described in the Supplementary materials. UK Biobank and the measures used for one-sample MR are described. |
|  | b) | Participants: Give the eligibility criteria, and the sources and methods of selection of participants. Report the sample size, and whether any power or sample size calculations were carried out prior to the main analysis | 18 to 20 and Table 1 | There were 462,690 participants from UKBB in the LSI GWAS and 941,280 participants from GSCAN (GWAS and Sequencing Consortium of Alcohol and Nicotine) in the drinks per week GWAS.  There were 148,726 cases and 965,732 controls from the Million Veteran Program, DIAMANTE and Biobank Japan for T2D. The continuous traits all used data from the Meta-Analyses of Glucose and Insulin-related traits Consortium (MAGIC), with 209,605 participants with data for fasting glucose, 158,550 with data for fasting insulin and 149,289 with data for HbA1c.  There were between 242,412 and 266,005 participants in our one-sample MR analyses see Supplementary Table S4). |
|  | c) | Describe measurement, quality control and selection of genetic variants | 18 to 19 | This is described in terms of p-value threshold used, the LD parameters, availability in outcome data and search for proxies if needed and finally in the harmonisation step description. |
|  | d) | For each exposure, outcome, and other relevant variables, describe methods of assessment and diagnostic criteria for diseases | 18 to 19 | GWAS measures are described briefly in the main paper with details in supplementary materials. Details for drinks per week, type 2 diabetes and HbA1c are given in the paper for UKBB data. |
|  | e) | Provide details of ethics committee approval and participant informed consent, if relevant | 25 | All studies that contributed to the exposure and outcome GWAS used in MR analyses had ethics approval and participant consent for their data to be used in genetic analyses. UKBB (data used in one-sample MR analyses) received ethics approval from the UK National Health Service Research Ethics Committee (11/NW/0382). |
| 5 | **Assumptions** | Explicitly state the three core IV assumptions for the main analysis (relevance, independence and exclusion restriction) as well assumptions for any additional or sensitivity analysis | 5 | Box 1 in the introduction |
| 6 | **Statistical methods: main analysis** | Describe statistical methods and statistics used |  |  |
|  | a) | Describe how quantitative variables were handled in the analyses (i.e., scale, units, model) | 18 to 24 | Drinks per week and HbA1c were log transformed, the units for each MR analysis are described at relevant points in the methods and the models used within the two-sample and one-sample MR sections, including sensitivity analyses. |
|  | b) | Describe how genetic variants were handled in the analyses and, if applicable, how their weights were selected | 18 to 19 | See above for selection of genetic variants |
|  | c) | Describe the MR estimator (e.g. two-stage least squares, Wald ratio) and related statistics. Detail the included covariates and, in case of two-sample MR, whether the same covariate set was used for adjustment in the two samples | 22 to 25 | The two-sample MR methods of IVW, MR-Egger, SIMEX adjusted MR-Egger, weighed median, MR PRESSO and GSMR are described in the methods. The one-sample MR used a 2SLS approach and covariates are listed on pg 24. |
|  | d) | Explain how missing data were addressed |  | NA - missing data was not imputed etc |
|  | e) | If applicable, indicate how multiple testing was addressed |  | NA |
| 7 | **Assessment of assumptions** | Describe any methods or prior knowledge used to assess the assumptions or justify their validity | 19 to 22 | Details of two-sample MR approaches and assumptions tested are listed as per above. |
| 8 | **Sensitivity analyses and additional analyses** | Describe any sensitivity analyses or additional analyses performed (e.g. comparison of effect estimates from different approaches, independent replication, bias analytic techniques, validation of instruments, simulations) | 22 to 24 | See above for two-sample MR  For one-sample MR we also conducted analyses adjusting for genotyping chip and also analyses including and excluding individuals with type 2 diabetes where HbA1c was the outcome |
| 9 | **Software and pre-registration** |  |  |  |
|  | a) | Name statistical software and package(s), including version and settings used | 22 & 24 | All analyses were conducted in R (version 3.6.2). We conducted two-sample MR analyses using the TwoSampleMR package in R. One-sample MR analyses were conducted using the OneSampleMR and Applied Econometrics with R (AER) packages, respectively, in R. |
|  | b) | State whether the study protocol and details were pre-registered (as well as when and where) | 21 | We pre-registered the analysis plan for this study on the Open Science Framework in March 2021 (https://osf.io/ygucn). |
|  | **RESULTS** |  |  |  |
| 10 | **Descriptive data** |  |  |  |
|  | a) | Report the numbers of individuals at each stage of included studies and reasons for exclusion. Consider use of a flow diagram |  | NA |
|  | b) | Report summary statistics for phenotypic exposure(s), outcome(s), and other relevant variables (e.g. means, SDs, proportions) |  | For one-sample MR this is reported in Supplementary Table S4. |
|  | c) | If the data sources include meta-analyses of previous studies, provide the assessments of heterogeneity across these studies |  | NA |
|  | d) | For two-sample MR:  i.  Provide justification of the similarity of the genetic variant-exposure associations between the exposure and outcome samples  ii.  Provide information on the number of individuals who overlap between the exposure and outcome studies | 18 to 19 | Sample overlap was avoided by using non-overlapping samples. |
| 11 | **Main results** |  |  |  |
|  | a) | Report the associations between genetic variant and exposure, and between genetic variant and outcome, preferably on an interpretable scale |  | NA |
|  | b) | Report MR estimates of the relationship between exposure and outcome, and the measures of uncertainty from the MR analysis, on an interpretable scale, such as odds ratio or relative risk per SD difference | 8 to 12 | Two-sample and one-sample MR results are presented in the main text and supplementary Table S2. |
|  | c) | If relevant, consider translating estimates of relative risk into absolute risk for a meaningful time period |  | NA |
|  | d) | Consider plots to visualize results (e.g. forest plot, scatterplot of associations between genetic variants and outcome versus between genetic variants and exposure) | 8 to 12 | See Figures 1 to 4 for two-sample MR results |
| 12 | **Assessment of assumptions** |  |  |  |
|  | a) | Report the assessment of the validity of the assumptions | 8 to 12 | Results from sensitivity analyses assessing assumptions are reported throughout results and also presented in Supplementary Table S2 and Figures 1 to 4. |
|  | b) | Report any additional statistics (e.g., assessments of heterogeneity across genetic variants, such as *I^2^*, Q statistic or E-value) | 8 to 12 | Results for additional statistics for two-sample MR are reported throughout results and also presented in Supplementary Table S2. Supplementary Table S1 reports F statistics. |
| 13 | **Sensitivity analyses and additional analyses** |  |  |  |
|  | a) | Report any sensitivity analyses to assess the robustness of the main results to violations of the assumptions | 8 to 12 | Results from sensitivity analyses assessing assumptions are reported throughout results and also presented in Supplementary Table S2 and Figures 1 to 4. |
|  | b) | Report results from other sensitivity analyses or additional analyses | 8 to 12 | Results from sensitivity analyses assessing assumptions are reported throughout results and also presented in Supplementary Table S2 and Figures 1 to 4. |
|  | c) | Report any assessment of direction of causal relationship (e.g., bidirectional MR) |  | NA |
|  | d) | When relevant, report and compare with estimates from non-MR analyses |  | NA |
|  | e) | Consider additional plots to visualize results (e.g., leave-one-out analyses) |  | NA – due to the high number of analyses performed |
|  | **DISCUSSION** |  |  |  |
| 14 | **Key results** | Summarize key results with reference to study objectives | 13 | Main results from all analyses are summarised in the first paragraph of discussion. |
| 15 | **Limitations** | Discuss limitations of the study, taking into account the validity of the IV assumptions, other sources of potential bias, and imprecision. Discuss both direction and magnitude of any potential bias and any efforts to address them | 15 to 16 | We discuss non-linear effects of alcohol, selection bias in UKBB, generalisability and measurement error. |
| 16 | **Interpretation** |  |  |  |
|  | a) | Meaning: Give a cautious overall interpretation of results in the context of their limitations and in comparison with other studies | 13 to 16 | Our results are discussed in the context of other studies and the limitations of our study. |
|  | b) | Mechanism: Discuss underlying biological mechanisms that could drive a potential causal relationship between the investigated exposure and the outcome, and whether the gene-environment equivalence assumption is reasonable. Use causal language carefully, clarifying that IV estimates may provide causal effects only under certain assumptions |  | NA |
|  | c) | Clinical relevance: Discuss whether the results have clinical or public policy relevance, and to what extent they inform effect sizes of possible interventions |  | NA |
| 17 | **Generalizability** | Discuss the generalizability of the study results (a) to other populations, (b) across other exposure periods/timings, and (c) across other levels of exposure | 15 to 16 | See limitations |
|  | **OTHER INFORMATION** |  |  |  |
| 18 | **Funding** | Describe sources of funding and the role of funders in the present study and, if applicable, sources of funding for the databases and original study or studies on which the present study is based | 26 | See funding section, there were no roles of funders in the study. |
| 19 | **Data and data sharing** | Provide the data used to perform all analyses or report where and how the data can be accessed, and reference these sources in the article. Provide the statistical code needed to reproduce the results in the article, or report whether the code is publicly accessible and if so, where | 27 | Data access is described in the Data availability section and Supplementary Table S5. |
| 20 | **Conflicts of Interest** | All authors should declare all potential conflicts of interest | 27 |  |

This checklist is copyrighted by the Equator Network under the Creative Commons Attribution 3.0 Unported (CC BY 3.0) license.

1. Skrivankova VW, Richmond RC, Woolf BAR, Yarmolinsky J, Davies NM, Swanson SA, et al. Strengthening the Reporting of Observational Studies in Epidemiology using Mendelian Randomization (STROBE-MR) Statement. JAMA. 2021;under review.

2. Skrivankova VW, Richmond RC, Woolf BAR, Davies NM, Swanson SA, VanderWeele TJ, et al. Strengthening the Reporting of Observational Studies in Epidemiology using Mendelian Randomisation (STROBE-MR): Explanation and Elaboration. BMJ. 2021;375:n2233.
